# Supplementary figures and images for: Functionally non-redundant paralogs spe-47 and spe-50 encode FB-MO associated proteins and interact with him-8
Source: PLoS One. 2020 Dec 31;15(12):e0230939. doi: 10.1371/journal.pone.0230939 (PMC7774929; doi:10.1371/journal.pone.0230939)

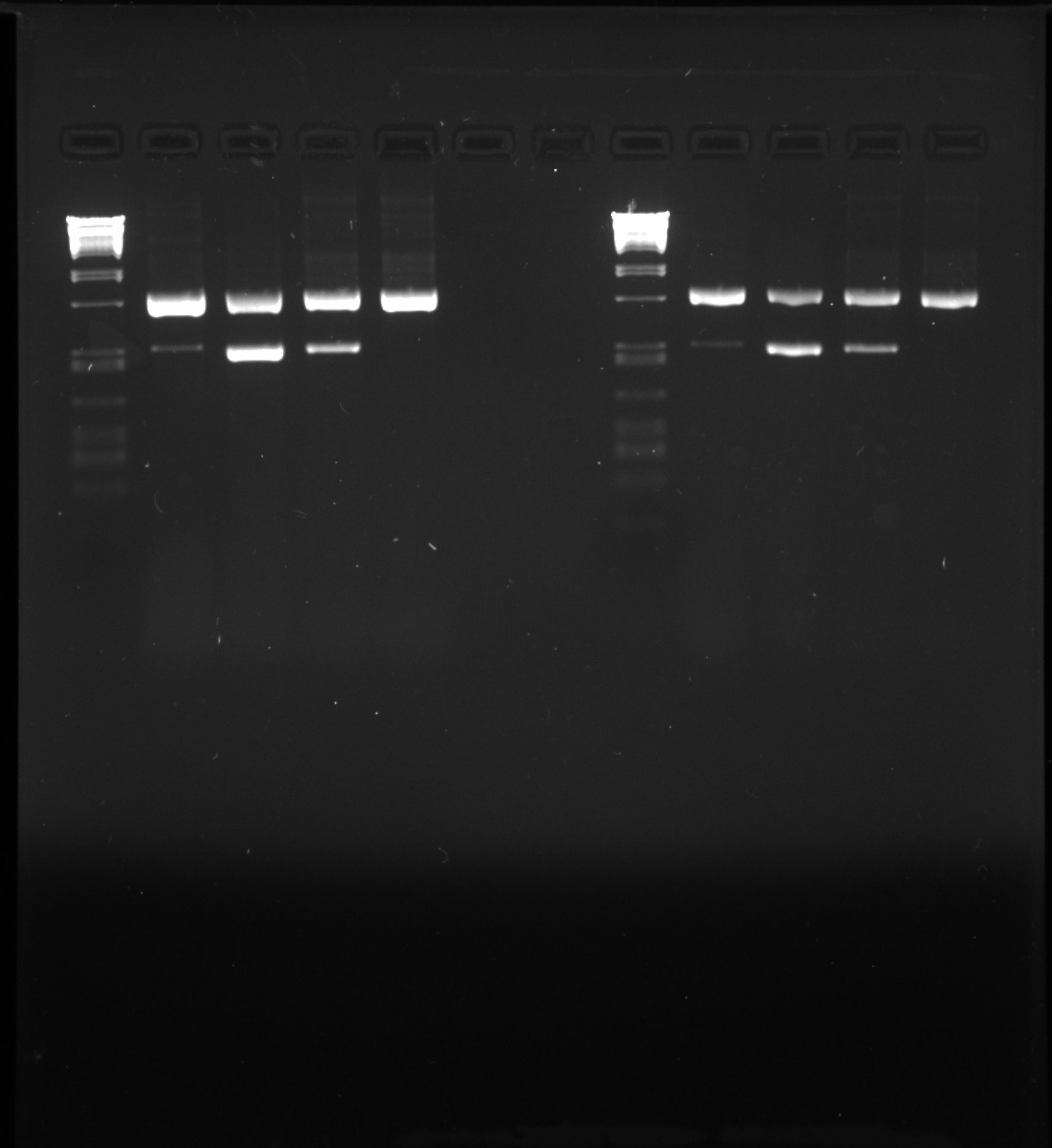

Supplement: S1 Raw image — The image used in Fig 2 is on the right. The set of bands to the left had a larger volume of RT-PCR products loaded into the wells than the set of bands that were included in Fig 2. (TIF) [file pone.0230939.s001.tif]
