# Supplementary material for: Functionally non-redundant paralogs spe-47 and spe-50 encode FB-MO associated proteins and interact with him-8
Source: PLoS One. 2020 Dec 31;15(12):e0230939. doi: 10.1371/journal.pone.0230939 (PMC7774929; doi:10.1371/journal.pone.0230939)
Supplement: S1 Dataset — (PDF) [file pone.0230939.s002.pdf]

Fertility Data

Data from Figure 4: All worms maintained at 25C from L4 stage onward

	spe-27(it132)	spe-47(hc198)	spe-47(hc198); spe-27(it132)	spe-50(zq26)	spe-50(zq26); spe-27(it132)	spe-47(hc198); spe-50(zq26)	N2
	0	58	1	258	0	45	132
	0	52	8	221	0	58	211
	0	35	1	183	0	60	221
	0	62	38	277	0	51	122
	0	36	44	249	0	59	157
	0	48	34	328	0	72	196
	0	51	3	247	0	92	105
	0	54	47	231	0	41	216
	0	55	31	278	0	59	198
	0	56	35	310	0	26	189
	0	46	0	283	0	58	213
	0	61	20	303	0	57	178
	0	47	38	322	0	70	164
	0	54	24	300	0	55	98
	0	51	29	311	0	52	
		64					
		64					
Mean	0.0	52.6	23.5	273.4	0.0	57.0	171.4
SEM	0.0	2.1	4.3	10.7	0.0	3.8	11.4

Data from Figure 5: worms reared at either 25C or 20C from L4 stage onward

	N2 (25C)	N2 (20C)	spe-47(zq27)(25C)	spe-50(ttTi4488)(25C)	spe-47(zq27); spe-50(ttTi4488)(25C)	him-8(e1489)(25C)	spe-50(ttTi4488); him-8(e1489)(25C)	spe-47(zq27); him-8(e1489)(25C)
	132	315	246	199	98	157	169	155
	211	355	323	225	124	156	121	199
	221	367	273	291	121	136	162	187
	122	312	223	341	97	148	139	179
	157	351	238	187	150	14	125	229
	196	380	208	156	94	177	148	223
	105	356	296	114	116	162	168	240
	216	390	227	208	119	136	139	169
	198	290	230	241	102	129	67	165
	189	379	263	224	156	132	71	189
	213	375	263	189	126	133	109	200
	178	435	179	205	103	195	138	215
	164	326	195	157	103	128	133	210
	98	470	226	180	117	107	91	205
		342	130	97	123	167		135
			266			128		
			222			160		
Mean	171.4	362.9	235.8	200.9	116.6	139.1	127.1	193.3
SEM	11.4	12.0	10.9	16.0	4.7	9.4	8.8	7.5

Data from Figure 5 continued

	spe-47(hc198); spe-50(ttTi4488); him-8(1489)(25C)	spe-47(zq27); spe-50(zq26); him-8(e1489)(25C)	spe-47(zq27); spe-50(ttTi4488); him-8(e1489)(25C)	spe-47(zq27); spe-50(ttTi4488); him-8(e1489)(20C)	spe-47(zq27); spe-50(ttTi4488); him-8(e1489) X him-8(e1489) males(25C)	him-5(e1490)(25C)	spe-50(ttTi4488); him-5(e1490)(25C)	spe-47(zq27); him-5(e1490)(25C)	spe-47(zq27); spe-50(ttTi4488); him-5(e1489)(25C)
	16	166	12	178	311	34	88	141	33
	16	176	0	201	435	110	48	123	19
	21	223	0	196	220	158	96	118	4
	21	123	14	193	373	103	82	133	37
	5	138	19	206	356	148	65	115	51
	3	193	19	113	341	31	78	147	55
	10	178	10	157	194	122	55	131	7
	19	187	15	178	80	124	57	141	102
	14	175	22	177	227	153	63	120	102
	15	199	41	104	299	59	41	127	91
	12	224	0	188		9	31	148	69
	10	110	34	162		46	61	135	74
	23	192		207		154	76	16	110
	17	162		210		94	66		105
	7	209		141		150			
		190							
Mean	13.9	177.8	15.5	174.1	283.6	99.7	64.8	122.7	61.4
SEM	1.6	8.1	3.7	8.6	32.9	13.3	4.8	9.4	10.0

Data from complementation tests. The data reported here are fertlity of the F1 offspring reared at 25C from the cross of the strain harboring ttTi4488 with the test gene (spe-7 or spe-18).

	spe-7(mn252)	spe-18(hc133)
	197	174
	225	223
	193	178
	246	155
	241	186
	222	204
	115	190
	187	225
	228	227
	125	195
		207
		116
mean	197.9	190.0
SEM	14.4	9.3
